# Retrograde filling material in periapical surgery: a systematic review

**DOI:** 10.4317/medoral.24262

**Published:** 2020-12-19

**Authors:** Anais Paños-Crespo, Alba Sánchez-Torres, Cosme Gay-Escoda

**Affiliations:** 1 DDS. Fellow of the Master of Oral Surgery and Implantology Degree Program, Medicine and Health Sciences Dental School, University of Barcelona, Spain; 2 DDS, MS. Master of Oral Surgery and Implantology. Associate Professor of Oral Surgery, Medicine and Health Sciences Dental School, University of Barcelona, Spain; 3 Researcher at the IDIBELL Institute. Barcelona, Spain; 4 MD, DDS, MS, PhD, EBOS, OMFS. Chairman and Professor of Oral and Maxillofacial Surgery, Medicine and Health Sciences Dental School, University of Barcelona, Spain; 5 Director of the Master Degree Program in Oral Surgery and Implantology (EFHRE International University/FUCSO); 6 Coordinator/Researcher at the IDIBELL Institute. Barcelona, Spain; 7 Head of the Department of Oral Surgery, Implantology and Maxillofacial Surgery, Teknon Medical Center, Barcelona, Spain

## Abstract

**Background:**

Periapical surgery focuses on the treatment of teeth with persistent periapical lesions when orthograde root canal treatment fails. Although MTA® is the gold standard material for retrograde filling, Biodentine® - a tricalcium silicate-based cement - has been proposed in order to resolve several of its limitations. A systematic review has been carried out to compare the physicochemical properties of Biodentine® versus MTA® as root-end filling material in periapical surgery.

**Material and Methods:**

An electronic search was conducted by two independent examiners during March 2020 in the Cochrane, PubMed-MEDLINE and Scopus databases. In addition, a manual search was made in specialized journals. Comparative human or in vitro studies that evaluated bond strength, the presence of marginal gap and sealing ability were included. No restriction on publication date was applied. Animal studies, clinical cases, cases series and expert opinions were excluded.

**Results:**

After analyzing 147 initially selected studies, 13 publications were included. Regarding bond strength, the studies seemed to evidence better performance of Biodentine® in both acidic and blood contaminated environments. In relation to the presence of marginal gap and sealing ability, the studies yielded contradictory results. According to some authors, the sealing ability of Biodentine® is greater than that of MTA® during the first 24 hours, though both materials prove equal after one week. Other authors recorded no significant differences.

**Conclusions:**

Considering the limitations and heterogeneity of the studies included, there is not sufficient evidence to confirm the clinical superiority of Biodentine® as a root-end filling material in periapical surgery.

** Key words:**Biodentine, MTA, retrograde filling, periapical surgery.

## Introduction

Traditionally, periapical surgery has been indicated for the treatment of teeth with periapical lesions when periapical disease persists despite orthograde root canal treatment (1-3). The success rate of this surgical procedure is approximately 91.9% (4,5), and is largely attribuTable to the preparation of a retrograde cavity that allows correct marginal adaptation of the material in all three dimensions of space - securing correct sealing of the root canal and avoiding microleakage of microorganisms into the periapical tissues (2,6-8).

Selection of the root-end filling material influences the final outcome of the procedure, since its main objective is to seal the apical region (9). The material used therefore should afford radiopacity, biocompatibility, antimicrobial activity, bioactivity and solubility, low cytotoxicity, good marginal sealing and adhesion to root dentin, compression resistance as well as dimensional stability, proper setting time, and biomimetic properties under static and functional conditions (2,3).

From a clinical point of view, tight sealing of the retrograde cavity is directly proportional to bacterial filtration and, consequently, to contamination of the periapical tissues. Therefore, the biomaterials used must have low porosity to reduce bacterial permeability and high wettability offering a positive correlation to sealing ability and penetration of the material into the dentinal tubules (6).

Multiple retrograde filling materials have been used in periapical surgery: silver amalgam, gutta-percha, zinc oxide eugenol (Super EBA®), glass ionomer, IRM and many more (10). Mineral trioxide aggregate (MTA®) stands out among all these materials, however. Initially, it was formulated for the treatment of root perforations in endodontics. However, over the years, the use of MTA® has expanded (7). It is currently considered the gold standard thanks to its bioactive properties, its osseoinductive and conductive power that favors tissue regeneration when it comes into contact with the pulp and periradicular tissues, its great sealing capacity, and its antimicrobial effect (1,3,8). Furthermore, thanks to its radiopacity, it is easily detecTable on control radiographs (6).

However, MTA® has a long setting time, poor handling properties, a high economic cost, low resistance to compression and flexion, and in addition may cause discoloration of the treated tooth (2,7,11,12).

In order to solve the clinical problems with MTA®, a wide range of bioceramic materials have been developed. In 2009, a new dental substitute, Biodentine® (Septodont, Saint Maur des Fossés, France) was introduced on the market. This material is a cement based on a powder component that contains 80.1% tricalcium silicate, 14.9% calcium carbonate and 5% zirconium dioxide as an opacifier. The other component is liquid and is a compound of calcium chloride, which accelerates setting, and a water-soluble polymer, which affords correct fluidity. This new biomaterial offers compression resistance similar to that of root dentin, and has certain advantages over its predecessors, since it reduces the setting time (10-12 minutes) without compromising biocompatibility or bioactivity, inducing the formation of apatite in phosphate solutions, and improving its manageability (8,13).

Taking into account that placement of the biomaterial occurs under conditions of humidity and possible infection, the low pH levels produced by periapical inflammation can modify its response. The bioactivity of the material depends on the presence of phosphates in tissue fluids, which will directly influence the microleakage rate and the root microstructure. In other words, measuring the bond strength of biocements is important to quantify their interaction with dentin (14). The need to carry out studies evaluating the physicochemical properties of the materials used is therefore justified. The available literature on MTA® is extensive, though few data are available on the behavior of Biodentine® in acidic environments (7).

The objective of this systematic review was to compare the physicochemical properties of Biodentine® and MTA® as root-end filling materials in periapical surgery.

## Material and Methods

This systematic review was conducted according to Preferred Reporting Items for Systematic Reviews and Meta-analyses (PRISMA) statement (15). The following PICO (Patients, Intervention, Comparison, Outcome) question was established: “In teeth subjected to periapical surgery, does Biodentine® offer better physicochemical properties versus MTA®, used as retrograde sealing materials, in terms of bond strength, presence of marginal space (gap) and sealing ability?”.

- Study selection criteria

Comparative human or experimental *in vitro* studies evaluating the physicochemical properties of both root-end filling materials in terms of bond strength (MPa), the presence of marginal space (gap)(µm) and sealing ability (µl / min, µl / h, µl / min / cmH2O) were included. Animal studies, clinical cases, case series and expert opinions were excluded. No restriction on language was applied.

- Search strategy

An electronic search was conducted by two independent examiners (APC, AST) during March 2020 in the Cochrane, PubMed-MEDLINE and Scopus databases. The search strategy used was ("Biodentine [tw]) AND (" MTA "OR" mineral trioxide aggregate "[tw] AND (" root end filling "[tw] OR (" retrograde filling [tw]) OR ("Periapical surgery" [tw] OR ("apical surgery" [tw]) OR ("endodontic surgical treatment" [tw]). In addition, a manual search was performed in the following journals: Journal of Endodontics, International Endodontic Journal, British Dental Journal, Clinical Oral Investigations, International Journal of Oral and Maxillofacial Surgery, International Medical Journal and Journal of Dental Research to identify those articles not included in the results of the electronic search.

- Selection of articles

First, the two independent examiners selected the articles by reading the title and the abstract. Then, the selected papers were read in full text. A third reviewer (CGE) resolved possible discrepancies. The Cohen kappa coefficient was calculated to assess agreement between the reviewers regarding the selected articles.

- Data extraction and quality analysis of the articles

In order to carry out the qualitative analysis, the data collected from the studies were distributed into Tables prepared for each outcome variable (bond strength, gap and sealing ability), including the name of the authors, the year of publication, sample size, the measurement method used, follow-up time, main outcomes and the level of scientific evidence according to the Strength of Recommendation Taxonomy (SORT) criteria (16).

## Results

**Figure 1:**
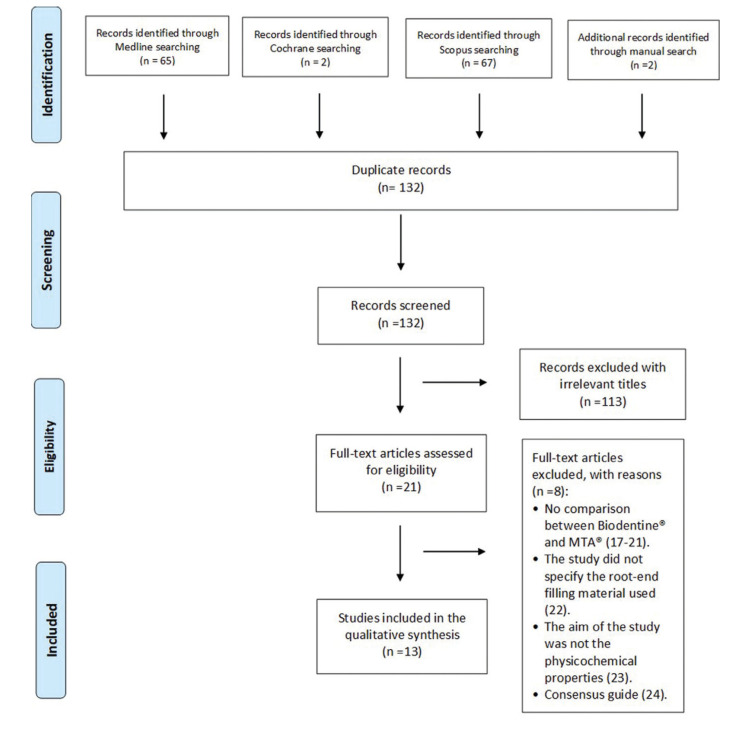
Flow chart of the articles following the PRISMA guidelines.

Fig. 1 displays the flow chart of the articles selected through the systematic review process following the PRISMA guidelines (15).

Of the 136 articles initially retrieved in the search, four duplicates were excluded; a total of 132 references were therefore reviewed. After analysis of the titles and abstracts, 21 articles were chosen for full-text evaluation. The authors agreed to exclude 8 of these articles on the grounds that they established no comparisons between Biodentine® and MTA® (17-21); they failed to specify the filling material used (22); the study objectives were unrelated to the physicochemical properties selected for our review (23); or because they constituted consensus guides (24). Thirteen articles were included (1-3,6-14,25) in the systematic review - all of them being *in vitro* studies corresponding to level 3 according to the SORT classification. The level of agreement between reviewers was excellent, with a kappa index of 1. Among the 13 selected articles, a total of 180 anterior teeth were treated with Biodentine® and 171 with MTA®.

Regarding bond strength, two of the four included studies, published by Akcay *et al*. (1) and Elnaghy *et al*. (11), reported statistically significant results in favor of Biodentine® (Table 1). Regarding the presence of gap, Ravichandra *et al*. (9) found it to be lower on surfaces filled with Biodentine®, while Biočanin *et al*. (6) recorded no statistically significant difference between the two materials. Soundappan *et al*. (3) obtained favorable results at 2 mm for MTA® (Table 2). Finally, sealing ability was the most controversial property, since Butt *et al*. (13) and Mazumdar *et al*. (10) recorded significant results in favor of Biodentine®, while Nabeel *et al*. (12) recorded data in favor of MTA®. However, both Agrafioti *et al*. (7) and Aydemir *et al*. (8), obtained favorable results for one of the two materials, according to the timing of the measurements (Table 3). Lastly, Shetty *et al*. (25) recorded no significant differences between Biodentine® and two types of MTA®.

**Table 1:**
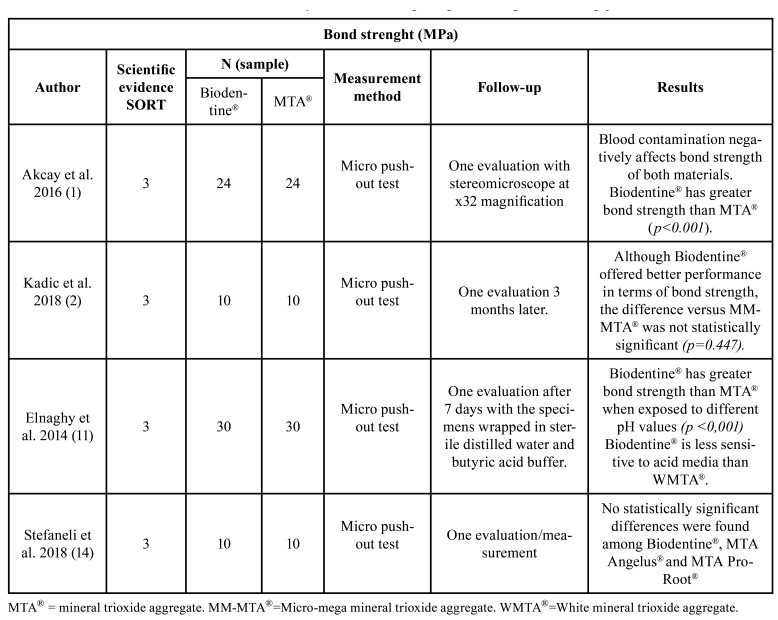
Characteristics of the studies selected for the systematic review regarding bond strength. MPa = Megapascals.

**Table 2:**
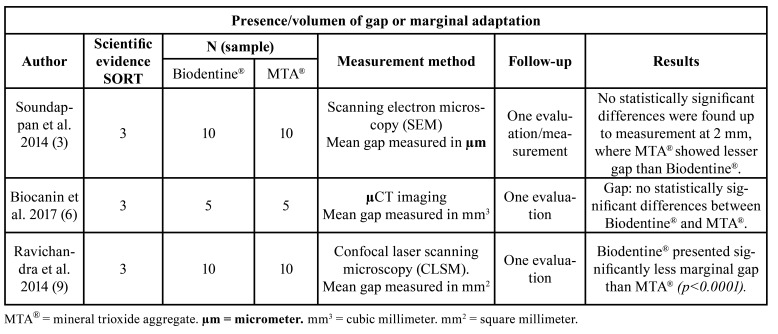
Characteristics of the studies selected for the systematic review regarding presence of microspace (gap).

**Table 3:**
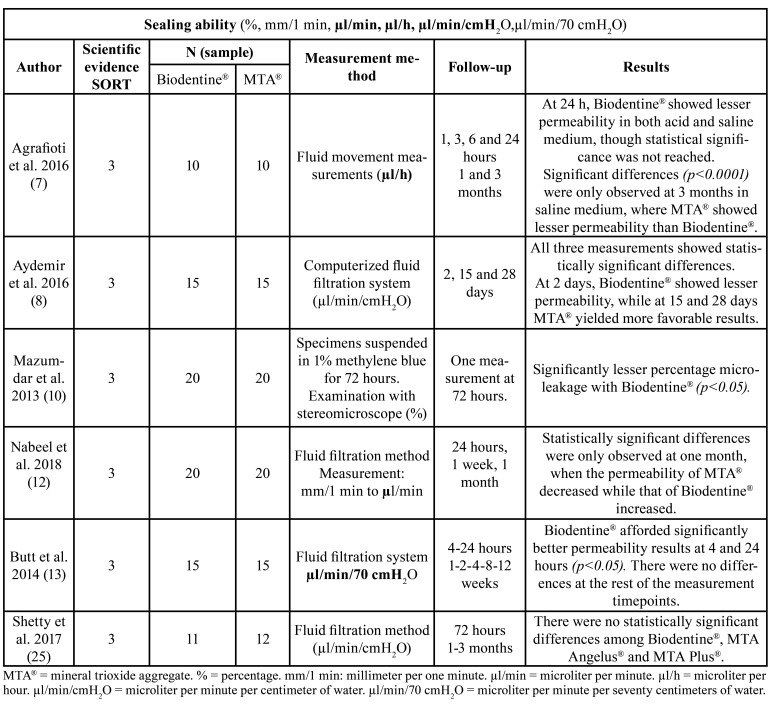
Characteristics of the studies selected for the systematic review regarding sealing ability.

## Discussion

Mineral trioxide aggregate (MTA®) remains the gold standard retrograde filling material in periapical surgery. At present, thanks to the creation of new biomaterials, emergent cements are capable of correcting some of the disadvantages of MTA® while maintaining its physicochemical properties. Biodentine® appears to be the most promising alternative in this regard. The main characteristic of tricalcium silicate-based cements is the precipitation of carbonate apatite in the presence of tissue fluids, followed by the formation of an interface that consolidates binding between the biomaterial and the root dentin - favoring bond strength, minimizing gap presence and reducing permeability.

Regarding the first of the mentioned properties, bond strength is defined as the bonding process between two surfaces with different molecular compositions as a consequence of chemical, physical or mechanical forces, and is influenced by the characteristics of the medium in which it is applied (14). In the study published by Akcay *et al*. (1), Biodentine® exhibited greater bond strength than MTA® in media characterized by blood contamination. These results are supported by the results of Elnaghy *et al*. (11), who found that although the bond strength of both materials decreased sequentially when exposed to increasingly acidic pH levels, Biodentine® offered better results. One of its main advantages is the acceleration of setting time, which is due to the presence of calcium chloride in its composition. Furthermore, even in the presence of blood, Biodentine® has great biomineralizing capacity capable of promoting the formation of crystalline structures at the dentin-cement interface, thereby improving resistance to torsion. In this respect, Biodentine® releases calcium-rich (Ca2+) products such as calcium phosphate, an element necessary to form these structures. Finally, the small size of its particles allows penetration into the dentinal tubules to a greater depth, improving micromechanical adhesion.

In the presence of tissue fluids such as blood, hydration of calcium silicate-based materials results in the formation of hydroxyapatite crystals and in the development of a hybrid interface between the root dentin and biomaterial. These two phenomena are altered in acidic environments because, although differently, the crystal structures of both materials change. Biodentine® has been shown to possess a structure comprising cubic crystals forming a honeycomb pattern, while the surface of MTA® appears more eroded, exhibiting a laminated, porous and stratified structure with a lack of formation of acicular or elongated needle-shaped crystals. Although the effects of these structural differences upon their behavior remains unclear, the authors affirm that they could be attributed to a lack of complete hydration of the material or to its poor setting (1,11).

On the other hand, Kadić *et al*. (2) considered Biodentine® to offer better bond strength than MTA®, though without reaching statistically significant differences. They justified the results differently, however. According to these authors, retention of the material and its physical properties are conditioned by the powder / liquid ratio, temperature and humidity, the amount of air trapped during mixing, and the size of the particles. Furthermore, since Biodentine® does not contain dicalcium silicate in its composition, it is much more homogeneous in terms of marginal adaptation, offering greater resistance to torsion.

On the other hand, in the study by Stefaneli-Marques *et al*. (14), both MTA Angelus® and MTA ProRoot® and Biodentine® offered similar results, without statistically significant differences. Nevertheless, the bond strength afforded by Biodentine® was seen to be accepTable, and was attributed to the small particle size – coinciding with the observations of Akcay *et al*. (1) and Kadić *et al*. (2) – the incorporation of calcium chloride in its composition to improve hydration, and the release of calcium and silicon ions that are absorbed by the dentin, improving the structure, chemical composition and resistance of the cement-dentin interface.

From a clinical point of view, the presence of a gap between the cementum and the root dentin is of vital importance for the success of periapical surgery, since it guarantees a "barrier effect" against bacterial filtration. According to Biočanin *et al*. (6), there are three material-related properties that could influence the quality of retrograde filling: micro- and nano-porosity, and wettability. The use of three-dimensional (3D) imaging allowed the authors to perform a volumetric reconstruction, showing that the samples filled with Biodentine® had fewer microspaces and were smaller before and after plasma immersion compared to MTA® and Fuji IX®. This agrees with the findings of Ravichandra *et al*. (9), where thanks to the presence of calcium carbonate in its composition, Biodentine® constitutes a powerful precursor of hydroxyapatite formation on porous surfaces. This bioactivity is capable of closing those pores that remained open - preventing microleakage and the penetration of pathogens.

Despite the above, it must be kept in mind that the presence or not of a gap does not depend exclusively on the physicochemical characteristics of the material but also on the standardized protocol that determines the mode and time of mixing, as well as application in retrograde filling. In contrast, Soundappan *et al*. (3) studied marginal adaptation at two different levels, and only at 2 mm were the differences between the materials seen to be significant. The results evidenced lower mean gap values ​​for MTA®. The difference between the results obtained may be explained by the diversity of the methods used, since Biočanin *et al*. (6) employed microtomography, while Ravichandra *et al*. (9) used confocal microscopy, and Soundappan *et al*. (3) made use of scanning electron microscopy. Despite this, the lower porosity of Biodentine® is beneficial in humid environments such as the surgical field in periapical surgery (26).

Sealing ability (Table 3) is influenced by the porosity and hydrophilic capacity of the retrograde filling material. Taking into account that Biodentine® is denser and less porous, its transmission of fluids is minimal (27). Accordingly, in the study published by Butt *et al*. (13), statistically significant differences were obtained at four and 24 hours favorable to Biodentine® that can be explained by alkalinization of the medium, resulting in the release of calcium hydroxide, due to the greater biomineralizing capacity of Biodentine® versus MTA® - since the interface it forms with dentin is much broader and richer in calcium and silicon, and because its main component (calcium silicate) interacts with the phosphate ions in saliva, favoring the formation of apatite deposits with a high sealing capacity. Furthermore, it is important to highlight that Biodentine® sets faster than MTA®, which also seems to justify the results obtained. No differences were found between the two materials at the other follow-up timepoints.

Similar results were obtained by Mazumdar *et al*. (10), who found Biodentine® to offer the lowest permeability rate (10%) compared with white MTA® (30%) and grey MTA® (25%) at measurement 72 hours after immersion of the sample in 1% methylene blue. This was largely explained by the formation of a smear rich in calcium and silicon ions at the cement-dentin interface, as already described by Stefaneli-Marques *et al*. (14).

In contrast to the above, Nabeel *et al*. (12) obtained measurements after the first 24 hours and at one week and one month, with statistically significant differences being observed at 30 days. Throughout the measurements, the permeability of MTA® was reduced, while in contrast that of Biodentine® increased due to the formation of a calcium silicate gel (Ca2OHSi), which in addition to increasing the pH value, precipitates upon the cement particles.

Continuing with the analysis, Shetty *et al*. (25) found no statistically significant differences between Biodentine® versus MTA-Plus® and MTA-Angelus®. At 72 hours, the permeability of all three materials was similar. In contrast, at one and three months, the permeability of Biodentine® increased while that of MTA-Angelus® decreased. In other words, over the long term, MTA-Angelus® offered better sealing capacity than Biodentine®, and these two materials in turn afforded better results than MTA-Plus®. The results corresponding to the first measurement are justified by the short setting time of Biodentine® (12 minutes), as already indicated by Nabeel *et al*. (12), its hydrophilic naturalization and its expansion when setting.

The discrepancies in the results obtained with these two materials could be due to the timing of the analyses. In the study by Agrafioti *et al*. (7), permeability measured at 24 hours was lower for Biodentine® in both acidic and non-acidic environments. After three months, in a saline environment, MTA® obtained better results than Biodentine®. However, in acidic medium, no statistically significant differences were found between the two materials. These opposite results can be justified by the setting time, since Biodentine® sets faster than MTA®. Despite this, it has been shown that in acidic environments both materials experience different changes in their microstructure, which is consistent with the observations of Akcay *et al*. (1) and Elnagy *et al*. (11). The authors therefore do not recommend the preferential use of one cement or the other in environments of this kind.

Lastly, in the study by Aydemir *et al*. (8), significant differences in permeability were observed for Biodentine® and MTA® at days 2, 10 and 28. At the first timepoint, the results were favorable to Biodentine®, though on days 10 and 28 the findings were favorable to MTA®, with the lowest filtration rate. This supports the data published by Nabeel *et al*. (12) and Shetty *et al*. (25), who attributed these results to the longer setting time of MTA® and its subsequent consolidation.

The main limitations of the presented results are due to the fact that the included *in vitro* studies involved different follow-up times and different measurement methods, thus resulting in a lack of homogenization. Clinical studies in humans are needed to assess the success of periapical surgery using these two materials, taking into account periapical bone regeneration and the absence of clinical and radiological signs and symptoms.

## Conclusions

Considering the heterogeneity of the results obtained in the included studies, it can be concluded that there is a lack of scientific evidence regarding the superiority of tricalcium silicate over mineral trioxide aggregate as a root-end filling material in periapical surgery. Randomized clinical trials are therefore needed to determine whether Biodentine® is an accepTable clinical alternative to MTA®.
